# The future is bright, the future is biotechnology

**DOI:** 10.1371/journal.pbio.3002135

**Published:** 2023-04-28

**Authors:** Richard Hodge, on behalf of the PLOS Biology staff editors

**Affiliations:** Public Library of Science, San Francisco, California, United States of America and Cambridge, United Kingdom

## Abstract

As PLOS Biology celebrates its 20th anniversary, our April issue focuses on biotechnology with articles covering different aspects of the field, from genome editing to synthetic biology.

## Teaser

As *PLOS Biology* celebrates its 20^th^ anniversary, our April issue focuses on biotechnology with articles covering different aspects of the field, from genome editing to synthetic biology. With them, we emphasize our interest in expanding our presence in biotechnology research.

This article is part of the *PLOS Biology* 20th Anniversary Collection.

Biotechnology is a revolutionary branch of science at the forefront of research and innovation that has advanced rapidly in recent years. It is a broad discipline, in which organisms or biological processes are exploited to develop new technologies that have the potential to transform the way we live and work, as well as to boost sustainability and industrial productivity. The new tools and products being generated have a wide range of applications across various sectors, including medicine, agriculture, energy, manufacturing and food.

*PLOS Biology* has traditionally published research reporting significant advances across a wide range of biological disciplines. However, our scope must continue to evolve as biology increasingly becomes more and more applied, generating technologies with potentially game-changing therapeutic and environmental impact. To that end, we recently published a collection of magazine articles focused on ideas for green biotechnologies that could have an important role in a sustainable future [1], including how to harness microbial photosynthesis to directly generate electricity [2] and using microbes to develop carbon “sinks” in the mining industry [3]. Moreover, throughout this anniversary year we are publishing Perspective articles that take stock of the past 20 years of biological research in a specific field and look forward to what is to come in the next 20 years [4]; in this issue, these Perspectives focus on different aspects of the broad biotechnology field—synthetic biology [5] and the use of lipid nanoparticles (LNPs) for the delivery of therapeutics [6].

One fast moving area within biotechnology is gene editing therapy, which involves the alteration of DNA to treat or prevent disease using techniques such as CRISPR-Cas9 and base editors that enable precise genetic modifications to be made. This approach shows great promise for treating a variety of genetic diseases. Excitingly, promising phase I results of the first in vivo genome editing clinical trial to treat several liver-related diseases were reported at the recent Keystone Symposium on Precision Genome Engineering. This issue of *PLOS Biology* includes an Essay from Porto and Komor that focuses on the clinical applications of base editor technology [7], which could enable chronic diseases to be treated with a ‘one-and-done’ therapy, and a Perspective from Hamilton and colleagues that outlines the advances in the development of LNPs for the delivery of nucleic acid-based therapeutics [6]. LNPs are commonly used as vehicles for the delivery of such therapeutics because they have a low immunogenicity and can be manufactured at scale. However, expanding the toolbox of delivery platforms for these novel therapeutics will be critical to realise their full clinical potential.

Synthetic biology is also a rapidly growing area, whereby artificial or existing biological systems are designed to produce products or enhance cellular function. By using CRISPR to edit genes involved in metabolic pathways, researchers can create organisms that produce valuable compounds such as biofuels, drugs, and industrial chemicals. In their Perspective, Kitano and colleagues take stock of the technological advances that have propelled the “design-build-test-learn” cycle methodology forward in synthetic biology, as well as focusing on how machine-learning approaches can remove the bottlenecks in these pipelines [5].

While the potential of these technologies is vast, there are also concerns about their safety and ethical implications. Gene editing, in particular, raises ethical concerns, as it could be used to create so-called “designer babies” with specific traits or to enhance physical or mental capabilities. There are also concerns about the unintended consequences of gene editing, such as off-target effects that could cause unintended harm. These technologies can be improved by better understanding the interplay between editing tools and DNA repair pathways, and it will be essential for scientists and policymakers to be cautious and work together to establish guidelines and regulations for their use, as outlined at the recent International Summit on Human Genome Editing.

Basic research has also benefitted from biotechnological developments. For instance, methodological developments in super-resolution microscopy offer researchers the ability to image cells at exquisite detail and answer previously inaccessible research questions. Sequencing technologies such as Nanopore sequencers are revolutionising the ability to sequence long DNA/RNA reads in real time and in the field. Great strides have also been made in the development of analysis software for structural biology purposes, such as sub-tomogram averaging for cryo-EM [8]. The rate of scientific discovery is now at an unprecedented level in this age of big data as a result of these huge technological leaps.

The past few years has also seen the launch of AI tools such as ChatGPT. While these tools are increasingly being used to help write students homework or to improve the text of scientific papers, generative AI tools hold the potential to transform research and development in the biotechnology industry. The recently developed language model ProGen can generate and then predict function in protein sequences [9], and these models can also be used to find therapeutically relevant compounds for drug discovery. Protein structure prediction programs, such as AlphaFold [10] and RosettaFold, have revolutionized structural biology and can be used for a myriad of purposes. We have recently published several papers that have utilized AlphaFold models to develop methods that determine the structural context of post-translational modifications [11] and predict autophagy-related motifs in proteins [12].

The future of biotechnology is clearly very promising and we look forward to being part of the dissemination of these important new developments. Open access science sits at the core of our mission and the publication of these novel technologies in *PLOS Biology* can help their widespread adoption and ensure global access. As we look forward during this year of celebration, we are excited that biotechnology research will continue to grow and become a central part of the journal. The future is bright and the future is very much biotechnology.
